# A Retrospective Observational Study on Post-Pandemic Effects of Endogenous and Exogenous Factors on Prematurity in Pregnant Women Under 18 Years of Age

**DOI:** 10.3390/healthcare13020197

**Published:** 2025-01-19

**Authors:** Florin Țovîrnac, Alina Mihaela Călin, Eva Maria Elkan, Nicoleta Andreea Țovîrnac, Valentin Marian Antohi, Alexandru Nechifor

**Affiliations:** 1 Clinic Surgical Department, Dunarea de Jos University of Galati, 800008 Galati, Romania; tovarnacf@yahoo.com (F.Ț.); alina.calin@ugal.ro (A.M.C.); andreeatovarnac@yahoo.com (N.A.Ț.); 2 Morphological and Functional Sciences Department, Dunarea de Jos University of Galati, 800008 Galati, Romania; cojocarumariaeva@yahoo.com; 3 Department of Business Administration, Dunarea de Jos University of Galati, 800008 Galati, Romania; 4 Department of Medical Clinical, Dunarea de Jos University of Galati, 800008 Galati, Romania; alexandru.nechifor@ugal.ro

**Keywords:** preterm births, APGAR score, adolescent pregnancies, education, vices, hypertension, diabetes

## Abstract

**Background/Objectives:** This research investigates the impact of exogenous and endogenous factors on fetal health in pregnant women under the age of 18, with a special focus on the influence of educational level, adherence to vices (smoking and alcohol), comorbidities (diabetes and hypertension), and poor sanitary conditions. **Methods:** The study uses retrospective data collected from a medical institution in the Southeast region of Romania, including a sample of 3639 births during the post-pandemic period (2022–2023). This period was considered to be a reference period for the study because, as a result of measures to combat the spread of COVID-19 disease in the pandemic, there was an increase in the birth rate among patients under 18 years of age. The APGAR clinical score was evaluated at 5, 10, and 20 min after birth and measured on an inverted scale to reflect the increased risk to fetal health. **Results:** The results indicate that lack of education is a significant exogenous factor associated with an increased risk of preterm births and a lower APGAR score. Additionally, adherence to vices is more pronounced among pregnant women with low educational levels and smoking and alcohol consumption negatively impact fetal health. Regarding comorbidities, diabetes did not significantly affect the short-term APGAR score, while hypertension had a complex effect, though medical interventions mitigated the associated risks. **Conclusions:** The conclusions of the research emphasize the need for appropriate educational and medical interventions to reduce the risks associated with preterm births and newborn health in adolescent pregnancies, especially in disadvantaged environments. The study suggests future research directions to expand the analysis to other geographical regions and for long-term monitoring of newborn health.

## 1. Introduction

Preterm birth is a major global public health challenge with significant implications for maternal and neonatal health, particularly among vulnerable groups. According to the literature [1], as well as guidelines developed by the American College of Obstetricians and Gynecologists [2] and the National Institute for Health and Care Excellence [3], preterm birth continues to be a global public health problem, with a particularly severe impact on pregnant adolescents under the age of 18, whose physiological and socioeconomic vulnerabilities amplify the risks associated with pregnancy. Some authors [4] argued that the risk of dependency is higher in adolescent mothers compared to adult ones. Furthermore, pregnant adolescents, due to their clinical association with neurological or psychiatric pathologies, face an increased risk of psychoactive substance use. According to Varmaghani et al. [5], a significant number of adolescent mothers (16.1%) link anemia and preeclampsia to lifestyle factors, along with nutritional deficiencies, which impact fetal birth weight. Additionally, recent studies [6] showed that young mothers, due to the lack of consistent social benchmarks and a perception of an unstable financial future, develop what is called “unstable identities”, making them more vulnerable to psychoactive substance use, especially in the context of chronic depression. In the post-pandemic period, the risks associated with adolescent preterm pregnancies have intensified due to various exogenous factors, such as limited access to medical services [7], environmental stressors, and socioeconomic instability.

Adolescents face higher risks of preterm birth due to incomplete physical development [8], lack of adequate prenatal care [9], and often unstable social environments [10]. The COVID-19 pandemic also brought additional challenges, creating a context of health uncertainty and restricting access to routine medical care, which amplified the risk of preterm births [11]. In addition to physiological and socioeconomic factors, young mothers often lack the resources needed to manage pregnancy properly [12]. Limited access to reproductive health education and the lack of family and social support contribute to worsening the risks associated with preterm birth [13]. Exogenous factors, including exposure to toxic substances, also play a crucial role in influencing maternal health and pregnancy outcomes, especially among adolescents, who are more vulnerable to these factors [14,15,16]. During the pandemic and post-pandemic periods, healthcare demands increased, while access to essential medical services, including prenatal monitoring, was reduced [17,18]. For pregnant adolescents, this lack of care led to an increased risk of complications, with preterm birth being a major concern. Isolation measures, fear of infection, and changes in access to services led many young women to avoid routine medical check-ups, hindering the early detection of pregnancy complications [19,20,21].

Another important factor in determining preterm birth is the influence of the social and family environment of the adolescent [22,23,24]. Families with low incomes, living in precarious conditions, may be unable to provide the necessary support for a healthy pregnancy. Additionally, stress caused by dysfunctional family relationships or the responsibilities placed on young mothers contributes to increased emotional and physical tension, negatively affecting maternal and fetal health. Pregnant adolescents are often exposed to additional risk factors such as smoking [25,26,27,28], alcohol consumption [29,30,31,32,33], and other harmful behaviors [34,35,36,37], which can worsen the mother’s health and influence fetal development. Studies show that these behaviors are more frequent among adolescents, often as a response to psychological stress and social pressure [38,39,40,41]. The pandemic exacerbated these behaviors through social isolation and additional emotional difficulties caused by imposed restrictions. The pandemic also amplified social and health inequalities, creating an environment conducive to the escalation of domestic violence, which in turn negatively affected the health of pregnant women and their children [42,43,44,45].

Exogenous factors, such as pollution and poor living conditions, also play an important role in increasing the risk of preterm birth. Studies have shown that exposure to fine airborne particles from pollution can negatively affect fetal health, leading to intrauterine growth restriction and preterm birth [46,47,48,49]. Adolescents living in areas with high pollution are more exposed to these risks. To address these challenges, it is essential to implement public health policies that support pregnant adolescents. These should include access to free and quality medical care, reproductive health education, and psychological support. Awareness campaigns are also needed to address exogenous risk factors and promote a healthy lifestyle among young mothers [50,51,52]. The impact of exogenous factors on the health of pregnant adolescents has been amplified in the post-pandemic period, creating an environment conducive to an increase in the incidence of preterm births. Efficient management of these risks requires a multidimensional approach. The aim of the study is to investigate the influence of exogenous factors, such as education level, addictive behaviors (smoking and alcohol consumption), and sanitary conditions, on fetal health in pregnant women under 18, assessed by the APGAR clinical score. The study also aims to examine the role of comorbidities (diabetes and hypertension) and how they affect the risk of preterm births and the health of newborns. Through this analysis, the research seeks to identify key factors contributing to fetal health risks in pregnant adolescents and provide recommendations for improving medical and educational interventions.

The study spans the post-pandemic years of 2022 and 2023. The study aims to assess the influence of exogenous factors—including educational level, adherence to vices (smoking and alcohol), sanitary conditions, and endogenous factors such as comorbidities (diabetes and hypertension)—on the risk of preterm births and the clinical APGAR score in pregnant women under the age of 18.

The novelty of the research lies in its comprehensive approach to the impact of exogenous and endogenous factors on fetal health in pregnant adolescents under 18, in a post-pandemic context. The study specifically analyzes the influence of lack of education on the risk of preterm birth and the APGAR clinical score, integrating relevant variables such as adherence to vices (smoking and alcohol), sanitary conditions, and comorbidities (diabetes and hypertension). An element of originality is the use of an inverted scale for measuring the APGAR score, offering a new perspective on the risks associated with fetal health. Additionally, the research highlights the differences between the short-term impact of hypertension and the long-term impact of diabetes on the health of newborns in adolescent pregnancies, a dimension that has been explored little in the specialized literature.

## 2. Materials and Methods

This research represents a retrospective observational study conducted using medical records from the “Braila County Emergency Clinical Hospital, Maternity Building D”, which comprises university clinical departments providing medical care, conducting educational activities, medical scientific research, and continuous education. The study examines how exogenous factors (education, smoking, alcohol use, sanitary conditions) and endogenous factors (diabetes, hypertension) influenced preterm birth risks and APGAR scores in pregnant women under 18 during 2022–2023.

### 2.1. Study Design and Data Collection

This retrospective study analyzed data from patient medical records, focusing on a sample of 3639 registered births, categorized into two cohorts: an observational group of 3585 adult women (19 years and older), including 231 cases of preterm birth (i.e., patients with gestational age less than 37 weeks and birth weight less than 2500 g, without twin pregnancies), and a target group comprising 54 adolescent mothers (<18 years old) who experienced preterm births. The retrospective design allowed for the identification of associations between endogenous factors, exogenous factors, and neonatal outcomes based on already documented clinical observations.

Inclusion criteria used in the case of the study are as follows: pregnant women who delivered at the hospital during 2022–2023 and had complete medical records, including APGAR scores and information on comorbidities and lifestyle factors. Participants were included regardless of socioeconomic status or living environment.

Exclusion criteria used in the case of the study are as follows: pregnant women with incomplete medical records, or births occurring outside the defined study period, were excluded to maintain data consistency and reliability (59 cases). Additionally, 18 cases with missing APGAR scores or incomplete documentation of endogenous or exogenous factors were omitted.

### 2.2. Ethical Considerations

Ethical approval was obtained from the hospital’s ethics committee prior to data collection (Institution: Ministry of Education of Romania, Dunarea de Jos University of Galati, 14.03.2024, Ethics Committee (CEU) number: CEU DECISION number 5). To ensure patient confidentiality, all data were anonymized through a coding system, where identifiable information was replaced with unique, non-identifiable codes. This study adhered to the principles outlined in the Declaration of Helsinki, emphasizing the rights and well-being of participants. Additionally, patient data were securely stored and accessed only by authorized researchers to minimize risks of breaches in confidentiality.

### 2.3. Variables and Measurements

Key variables (see Appendix A) included structural information of preterm labor/delivery/birth maternal age (Age), education level (Studies), living environment (EnvUR), type of childbirth (TypeB), information about exogenous factors such as adherence to vices (Vices), tobacco using (Smoking), and alcohol consumption (Alcohol). The APGAR score, an established measure of neonatal health, was recorded at 5 min post-birth (APGAR5), 10 min post-birth (APGAR10), and 20 min post-birth (APGAR20), with an inverted scale used to highlight risks. Additional variables included the presence of comorbidities (endogenous factors) such as diabetes mellitus (DM) and hypertension (HBP).

The ages of the pregnant women ranged from 12 to 45 years, and the patients were divided into 3 age categories (356 individuals aged 12 to 18 years, 3136 individuals aged 19 to 39 years, and 148 individuals over 40 years). The pregnant women came from the South-East region of Romania, including Braila, as well as neighboring counties Tulcea and Galati. Of the 3639 pregnant women included in the observational study, 1925 lived in urban areas, while 1714 lived in rural areas.

The patients in the target group (E) and observational group (P) are pregnant women with preterm births, with those in group E being mothers under the age of 18. Education level: 771 individuals have higher education, 1495 have secondary education, 1084 have completed middle school, 252 individuals have no education, and 38 have undeclared education levels.

A total of 1277 individuals had vaginal births, while 2362 had cesarean sections. Additionally, 339 individuals had comorbidities related to diabetes (DM), and 389 individuals had hypertension (HBP). Regarding addictive behaviors, 996 individuals reported smoking, 991 individuals reported alcohol consumption, and 857 individuals reported combined addictive behaviors. The clinical APGAR score received at birth was also analyzed at three distinct time points (5 min after birth, 10 min after birth, and 20 min after birth).

### 2.4. Statistical Analysis

Data analysis was performed using SPSS (version 26) with multiple linear regression models developed to explore the associations between exogenous factors and neonatal outcomes. Descriptive statistics were calculated for both groups, and comparisons were drawn to evaluate differences in maternal and neonatal health indicators. Special attention was given to identifying patterns within the experimental group of adolescent mothers, particularly regarding the compounded effects of socioeconomic and health-related factors.

## 3. Results

Descriptive statistics for the analyzed samples are presented in Table 1.

Table 1 categorizes the 3639 subjects, specifically all the pregnant women who gave birth at the Braila County Emergency Clinical Hospital, Building D, in the post-pandemic years 2022–2023, into three distinct categories: pregnant women with full-term births (3354 individuals), pregnant women over the age of 18 who gave preterm birth (231 individuals), and pregnant women under the age of 18 with preterm births (54 individuals).

Mothers under 18 years old (group E) represent a distinct group, predominantly from rural areas (54% compared to 46% for the observational group P). This suggests an association between the rural environment and the increased incidence of preterm births among teenagers, indicating more limited access to adequate medical care and social support compared to mothers from urban areas. The majority of mothers under 18 years old (group E) have a low education level, corresponding to the middle school cycle (1–8 grades). The average level of education in this group is 2 (upper secondary education), significantly lower than the average of 3.39 (secondary education) in the observational group P, which consists of mothers aged 19–39 years. This reflects another important exogenous factor—the level of education—that may influence access to information about maternal health and the ability to make informed decisions during pregnancy.

Both groups, E (mothers under 18 years old) and P, have a similar proportion of cesarean births, which highlights that the mode of delivery does not differ significantly between the two groups. However, frequent surgical intervention can be associated with additional complications for adolescent mothers, especially in the context of a health condition weakened by exogenous factors. The percentage of adherence to smoking and alcohol is much higher among mothers in group E (57% for smoking and 31% for alcohol) compared to group P (19% and 20%, respectively). This emphasizes the impact of addictive substances on maternal and fetal health in pregnant teenagers. The adherence to cumulative vices (smoking and alcohol) is also higher in group E, which can exacerbate the risks of preterm birth and postnatal complications. The incidence of comorbidities such as diabetes and hypertension is higher among mothers under 18 years old (19% and 44%) compared to those in group P (16% and 21%). These conditions can significantly contribute to the risk of preterm birth and the poor health of newborns. Regarding the immediate health of newborns, the APGAR score at 5, 10, and 20 min is slightly lower in group E compared to group P (7.76 vs. 7.85 at 5 min).

This lower score may be an indicator of the negative influence of exogenous factors on the health of newborns from the adolescent mother group. Although the differences are relatively small, they reflect a higher vulnerability of these newborns. The standard deviation of the APGAR scores is higher for adolescent mothers (group E), which suggests a higher variability in the health condition of newborns in this group. This indicates an instability of the results related to their overall condition, reflecting a more pronounced influence of exogenous factors on their health. The analysis of the descriptive statistics of the groups shows that low educational level and rural environment are major factors negatively affecting maternal health and increasing the risk of preterm birth among adolescents, thus highlighting the need for public policies that improve access to education and prenatal care in disadvantaged rural communities. The data suggest a significant influence of exogenous factors on prematurity among mothers under 18 years old, emphasizing the need for specific interventions for this vulnerable group. Addictive behaviors, comorbidities, and lack of education are the main factors that exacerbate the risks of prematurity and negatively affect the health of the mother and child.

The dynamic econometric model of the APGAR prematurity risk was developed for both groups (E and P) using multiple linear regressions (Table 2), with the regression coefficients determined by the least squares method using SPSS version 26.

Interpretation of regression equations for groups of mothers under 18 years old (group E) and the observational group (group P), based on the APGAR score monitored at 5, 10, and 20 min (Table 2), is performed considering that the APGAR score is measured on an inverse scale (where 1 represents the lowest risk and an APGAR score of 10, and 10 represents the highest risk and an APGAR score of 1). Thus, we take into account that negative coefficients indicate a decrease in the risk associated with the APGAR score, while positive coefficients indicate an increase in the risk associated with the APGAR score. Thus, the dynamic econometric model of APGAR risk of prematurity for group E (mothers under 18 years) highlights the negative impact of alcohol adherence (+2.187 in the case of the APGAR score measured at 5 min), which indicates that alcohol has a negative effect, increasing the risk and leading to a lower APGAR score (closer to 1). For APGAR5, the significance of this coefficient is limited (*p* = 0.113). At 10 min after birth, alcohol adherence of mothers under 18 years is associated with an increase in risk (+4.105), with the coefficient being significant (*p* = 0.002) and indicating a worse condition than in the previous APGAR evaluation.

In the case of the evaluation at 20 min, maternal alcohol adherence (+4.272) significantly increases the risk (*p* = 0.001), indicating a negative influence on the newborn’s health. Unlike alcohol, smoking adherence of pregnant women under 18 years old does not generate significant effects (*p* = 0.472 for APGAR5, *p* = 0.427 for APGAR10, and *p* = 0.439 for APGAR20). These results are explained by reduced cumulative exposure to smoking and biological differences in nicotine metabolism. Thus, pregnant women under 18 years old tend to smoke for a shorter period compared to adult women in group P. Since the harmful effects of smoking on health, including fetal health, are often associated with prolonged exposure, the shorter exposure duration to smoking in adolescents may not generate as marked effects on the APGAR score. The influence of uncertainties in smoking effects is reflected in the combined indicator (vice adherence), which does not provide a relevant representation of the risk reflected by the APGAR score dynamics.

Analysis of regression Equations (1), (3), and (5) highlights that hypertension has clearly negative effects on the risk associated with a reduced APGAR score in pregnant women under 18 years old. In the case of the APGAR score measured at 5 min (+2.980), HPB significantly increases the risk (*p* = 0.004), being an exogenous factor that worsens the score value. For APGAR10 and APGAR20, hypertension (+2.395) slightly reduces its influence compared to the previous moment on the risk, being a significant factor (*p* = 0.011 for APGAR10 and *p* = 0.013 for APGAR20). Diabetes is not a significant risk factor for pregnant women under 18 years old in group E, which can be explained by the lower prevalence of diabetes in adolescents and the long-term cumulative negative effects of diabetes. Similar to the smoking situation, diabetes in adolescents can be overshadowed by the influence of other exogenous risk factors that have a more significant impact on fetal health. It is observed that under the impact of increased adherence to vices among pregnant women under 18 years, the incidence of comorbidities (diabetes mellitus and hypertension) increases, but their impact differs. The effects of diabetic pathology develop over time and do not significantly influence the clinical APGAR score, while hypertension acts as an imminent risk factor that has an immediate effect on the depreciation of the APGAR score.

The dynamic econometric model of APGAR risk of prematurity for group P highlights the significantly increased negative impact of alcohol adherence (+4.021 in the case of the APGAR score measured at 5 min compared to the value of the same coefficient in group E +2.187), indicating that long-term alcohol consumption amplifies its negative effect, significantly increasing the risk (*p* = 0.000 in group P compared to *p* = 0.113 in group E). At 10 min after birth, alcohol adherence of mothers with preterm births over 18 years of age is associated with an increase in risk (+4.050, close to the coefficient value for group E), with the coefficient being significant (*p* = 0.000) and indicating a worse condition than in the previous APGAR evaluation. In the case of the evaluation at 20 min, maternal alcohol adherence (+4.008) slightly delays the risk compared to previous moments, being significant for risk (*p* = 0.000), indicating a negative influence on the newborn’s health. Unlike the target group, smoking adherence of pregnant women over 18 years old with preterm births generates significant effects (*p* = 0.000 for APGAR5, APGAR10, and APGAR20). However, the coefficient value, as in the case of group E, is negative according to Equations (2), (4) and (6). This aspect denotes an inverse correlation of smoking addiction with the risk associated with a lower APGAR score.

Smoking can affect the mother’s general health and implicitly that of the fetus, but in certain contexts, it may not be the most determining factor in the APGAR score. Genetic factors, lifestyle, and access to prenatal care play a complex role in how smoking affects the newborn. Therefore, the negative coefficient suggests that in this specific population, smoking does not directly correlate with the risk of a very low APGAR score but with a relatively more stable condition of the newborn than might be anticipated due to other factors. Unlike the target group E, the influence of uncertainties in smoking effects is not reflected in the combined indicator (vice adherence), which provides a positive representation of the risk reflected by the APGAR score dynamics, but the coefficient values are not statistically significant (*p* > 0.05 in all three moments analyzed). Analysis of regression Equations (2), (4) and (6) highlights that hypertension maintains its positive effects on the risk for pregnant women over 18 years old with preterm births, but the significance coefficients exceed the error confidence threshold (*p* > 0.05 in all three moments analyzed). As in the case of group E, diabetes records an inverse correlation with the dependent variable, potentially overshadowed by the influence of other exogenous risk factors that have a more significant impact on the fetal health of the preterm newborn.

The dynamic econometric APGAR model for the risk of prematurity for the groups of pregnant women under 18 years old (E) and adult mothers (P) based on the APGAR scores at 5, 10, and 20 min generated the following results of statistical significance according to the data in Table 3.

The dynamic econometric APGAR model of prematurity risk for the groups of pregnant women under 18 years old (E) and adult mothers (P), based on APGAR scores at 5, 10, and 20 min, generated the following statistically significant results according to the data in Table 3.

The statistical results for group E (pregnant women under 18 years old with preterm births) at the three observation points of the APGAR score reveal that approximately 49% of the variation in the APGAR score at 5 min is explained by the predictive variables (smoking, alcohol, vices, diabetes, and hypertension). The F value is 9.093 (*p* = 0.000), which suggests that the model is statistically significant, meaning the predictive variables have a significant influence on the APGAR score. Poor sanitary conditions and low socioeconomic status contribute to adverse neonatal health outcomes, as reflected in lower APGAR scores, and the result of this model confirms that these factors contribute to the increased risk of poor fetal health for preterm infants. The predictive power of the model increases for the APGAR score monitored at 10 min, where 61.4% of the variation in the score is explained by the model, which is also maintained for the APGAR score monitored at 20 min, generating a statistical representativeness level of 61.2%. The F change values (15.250 and 15.130) and *p* = 0.000 indicate that the model is extremely significant. This observation confirms that the exogenous factors continue to destabilize the APGAR score, having a negative effect on the health of the newborn for pregnant women under 18 years old.

For pregnant women over 18 years old with preterm births (group P), the statistical results of representativeness and significance indicate a lower capacity to explain the variation in the APGAR score monitored at 5 min (approximately 28%). However, the model is significant (*p* = 0.000), showing that smoking, alcohol, vices, diabetes, and hypertension also influence the APGAR score in pregnant women over 18 years old. The predictive power of the model increases for the APGAR score monitored at 10 min, as in the target group, but the representativeness remains weak (below 32%). The model is significant (*p* = 0.000), and smoking, in particular, has a significant impact, which suggests that adherence to unhealthy lifestyle behaviors, including smoking and alcohol consumption, is more prevalent among adolescent mothers and exacerbates the risk of preterm birth and poor neonatal outcomes. Table 4 presents the result of the ANOVA test for validating the dynamic econometric APGAR preterm risk model.

The interpretation of Table 4 (ANOVA) for the APGAR scores at 5, 10, and 20 min confirms a higher level of statistical significance for the results obtained for group E (pregnant women under 18 years old) compared to the results obtained for group P (adult mothers), with the regression mean squares for group E being strictly higher than the regression mean squares for group P. This confirms that the lack of education and poor sanitary conditions affect the health of newborns. In group P (adult mothers), smoking and alcohol have a significant impact on the APGAR score, and hypertension has an immediate effect on the score, as suggested by the regression coefficients.

The interpretation of Table 5, which presents the Pearson correlations between the analyzed variables, was carried out considering that the APGAR score was measured on a reversed scale to highlight the increasing fetal health risk as the score decreases. In addition, the independent variables were scaled on a gradual risk-increasing scale (e.g., education level, with the rest of the variables presented in Appendix A). Positive correlation values indicate a positive relationship between risks, while negative values indicate an inverse relationship.

The correlation between “Studies” and “Premature” for group E (r = −0.313, *p* = 0.011) suggests that a lower educational level is associated with an increased risk of preterm births. This inverse relationship indicates that lack of education increases the risk of prematurity. The negative correlation between “Studies” and APGAR5 (r = −0.241, *p* = 0.040) suggests that a lower educational level is associated with a higher risk to fetal health, resulting in lower APGAR scores (reflecting higher risks). The same trend is observed for APGAR10 (r = −0.180, *p* = 0.096) and APGAR20 (r = −0.168, *p* = 0.112), although the correlations are less significant. Furthermore, the variable “EnvUR” (urban/rural environment) has a negative correlation with APGAR5 (r = −0.209, *p* = 0.064) in group E, suggesting that pregnant women from rural areas have a higher risk to fetal health, associated with lower APGAR scores. These correlations emphasize that lack of education and a disadvantaged environment contribute to a higher risk for newborn health. The positive correlation between “Studies” and smoking (r = 0.377, *p* = 0.002) and between “Studies” and alcohol (r = 0.541, *p* = 0.000) indicates that a lower educational level increases the likelihood of addictive behaviors.

Additionally, combined vices (smoking + alcohol) are positively correlated with lack of education (r = 0.403, *p* = 0.001). These correlations demonstrated that low education is a contributing factor to adherence to vices among pregnant women under 18. Diabetes (DM) has an insignificant negative correlation with APGAR scores (APGAR5: r = −0.181, *p* = 0.095), suggesting that diabetes does not significantly affect the APGAR score in group E. This result confirms that maternal comorbidities such as hypertension and diabetes have differential effects on neonatal health, with hypertension posing a more immediate risk to APGAR scores, while the effects of diabetes manifest over time. Hypertension (HBP) has a significant positive correlation with APGAR5 (r = 0.439, *p* = 0.000), APGAR10 (r = 0.437, *p* = 0.001), and APGAR20 (r = 0.434, *p* = 0.001) in group E, indicating that hypertension has the expected negative effect on the APGAR score.

## 4. Discussion

**Figure 1:**
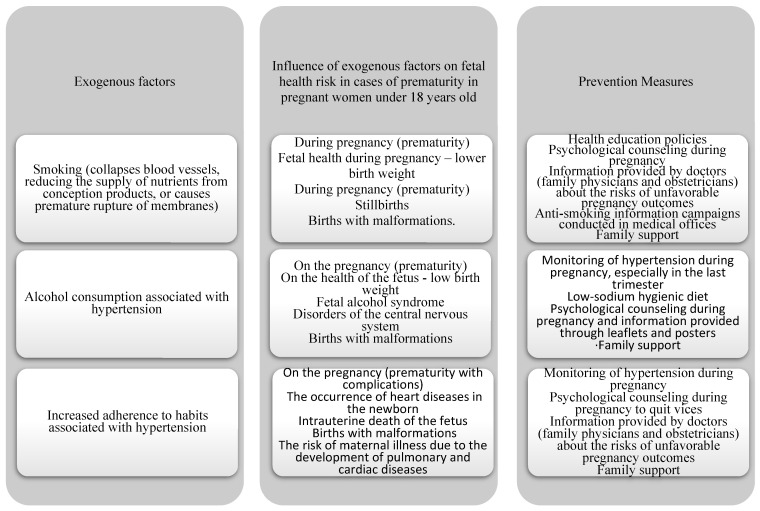
Clinical dashboard of fetal health risk prevention for pregnant women under 18 years old.

The results obtained allow for defining the following clinical dashboard for the prevention of fetal health risk in pregnant women under 18 years old (Figure 1).

Figure 1 illustrates the importance of continuous and personalized monitoring to prevent complications, as well as the need to implement specific strategies for the vulnerable group of pregnant women under 18 years old. Young pregnant women (under 18 years old) are a medically vulnerable group with increased risks to their health and that of the fetus. Figure 1 highlights exogenous factors, their influence on fetal health, and appropriate preventive measures. Smoking is a major exogenous risk factor with direct effects on blood vessels. Smoking interrupts the supply of nutrients needed by the fetus, leading to low birth weight and increased risk of premature rupture of membranes. Published studies [53,54,55] have shown that fetal exposure to nicotine contributes to severe complications. Other authors [56,57,58] have stated that premature rupture of membranes is a frequent consequence of maternal smoking. This practice is also associated with an increased incidence of maternal and neonatal infections and intrauterine growth restriction [59,60,61]. Another major risk factor is alcohol consumption during pregnancy, which is associated with hypertension, with serious consequences for both maternal and fetal health. Fetal alcohol syndrome is a well-documented complication, as detailed in the literature. Alcohol exacerbates hypertension, worsening the risk of preeclampsia and other complications, including structural abnormalities of the central nervous system and persistent cognitive dysfunction in the newborn [62,63,64]. Increased adherence to habits associated with hypertension is another determinant. Some studies [65,66,67] highlighted the negative impact of excessive salt intake and sedentary lifestyle on the health of pregnant women. These habits contribute to hypertension and risks of prematurity, leading to severe complications such as placental insufficiency and severe preeclampsia [68,69]. According to a 2018 study [70], researchers have demonstrated a causal relationship between high sodium dietary intake (which can affect brachial artery flow-mediated dilation) and the development of hypertension. In the literature, it is known that pre-existing hypertension in pregnancy is associated with an increased risk of gestational hypertension and preeclampsia [71]. Some authors, Bank et al. [72], estimated that sodium intake was not associated with the risk of developing a hypertensive disorder of pregnancy. However, there are contrary opinions in the literature that low sodium diets and regular blood pressure monitoring were associated with reduced risk of preeclampsia [73,74]. Also, in the recent literature, there are studies [75,76] confirming that the adoption of healthy diets can significantly contribute to the prevention of complications. Studies suggest that calcium and vitamin D supplementation may reduce the risk of pregnancy-induced hypertension [75,76].

Exogenous factors have a significant influence on the risks of prematurity and fetal disorders, with a particular impact on under-age mothers. During pregnancy, prematurity is common in underage mothers and is caused by both biological and socioeconomic factors. According to studies [77,78,79], these young mothers are at increased risk of preterm births, leading to low birth weight and other complications such as congenital malformations. Factors such as limited access to healthcare and insufficient social support exacerbate these risks. In cases of exposure to alcohol and smoking, the damage to fetal health is acute. Fetal alcohol syndrome and central nervous system disorders are among the most common documented effects. Studies have shown that alcohol-associated prematurity increases the risk of giving birth to a malformed and underweight baby [80,81]. Maternal smoking is also correlated with increased incidence of sudden infant death syndrome (SIDS) [82,83]. For the mother, the consequences include cardiovascular risks and lung disease. Studies in the literature highlight that pregnant adolescents are at significantly increased risk of developing hypertension and associated cardiovascular disease, emphasizing the need for appropriate medical monitoring and interventions [84,85,86]. Adolescents are also at higher risk of severe anemia, which worsens maternal health and negatively impacts fetal development.

Health education is essential to reduce the risks associated with pregnancy in underage mothers. Anti-smoking campaigns have been shown to be effective, and implementation of these programs in physicians’ offices can significantly reduce associated complications [87,88]. Education about proper nutrition and the importance of regular prenatal check-ups can help prevent complications [89,90]. Recent research [91,92] shows that tailored educational programs significantly increase awareness of the risks of smoking, and other studies support the importance of involving family physicians in prenatal education to reduce behaviors’ risk [93,94].

The research took into account that the risk associated with preterm pregnancies in minors increases in the absence of reproductive health education, [13,19,20,21]. The results of the research highlighted the need to improve reproductive health education through the adoption of public policies that favor access to specific health education for minors, more specifically the development of education and information activities in schools through specialized medical staff and the support of education and information activities in family doctors’ offices both through counseling and through educational materials displayed in the medical offices. Counseling plays an important role in preventing behaviors’ risk, with studies showing that counseling programs reduce harmful substance use during pregnancy [95,96]. Psychological support also helps in managing stress and anxiety, which are commonly associated with adolescent pregnancy. Family support is essential for adolescent mothers; family support reduces maternal stress and contributes to adherence to medical recommendations. Family involvement in medical education and monitoring is vital to prevent complications, and social support networks can provide additional resources to support pregnant adolescents in making healthy decisions [36,97].

These preventive measures, integrated into a system of continuous monitoring, offer a holistic approach to reducing the risks associated with pregnancy in under-age mothers and ensuring healthy fetal development. A multidisciplinary approach, including physicians, psychologists, and social workers, is essential to ensure the success of these interventions.

### 4.1. Limitations of the Study

The study focused on pregnant women from a single region of Romania (South-East), which may limit the generalization of the results at a national or international level. Cultural, economic, and social variables in other regions may influence the risk of preterm births and APGAR scores differently. The study did not analyze in detail other potentially important exogenous factors, such as psychological stress levels, social support, or socioeconomic conditions, which could influence the health of pregnant women and newborns. Additionally, the APGAR score provides a snapshot of the newborn’s health in the short term (5, 10, and 20 min after birth), but the research did not track the long-term development of prematurely born children to assess the subsequent impact of risk factors.

### 4.2. Future Research Directions

The authors aim to extend the study to other regions and populations to test the relevance and applicability of the conclusions in other sociocultural and economic contexts. Additionally, a longitudinal study would allow researchers to track the health and development of prematurely born children to assess the long-term impact of low education, addictive behaviors, and maternal comorbidities on the children. These future directions will provide a deeper and more nuanced understanding of the factors influencing the health of adolescent pregnant women and newborns, contributing to the development of effective intervention strategies.

## 5. Conclusions

The conclusions of the research reflect the major impact that exogenous and endogenous factors have on fetal health in pregnant women under 18, within the context of preterm births and the APGAR clinical score. The models for pregnant women under 18 years old explain the variation in the APGAR score much better than those for adult mothers, indicating a stronger influence of exogenous risk factors in adolescents. Lack of education is a determining factor for preterm births, with the study demonstrating that lower educational levels significantly increase the risk of preterm births in adolescent pregnant women. Pregnant women with lower education levels are at a higher risk of giving preterm birth, which is associated with living conditions and limited access to information and medical care.

Adverse sanitary and social factors negatively influence the APGAR score. We have shown that poor sanitary conditions, associated with rural environments and lack of education, destabilize the health of newborns, resulting in lower APGAR scores. Pregnant women from rural areas, with limited access to adequate prenatal care, are at a higher risk of giving birth to children with health problems immediately after birth. Adherence to unhealthy habits contributes to the deterioration of fetal health, demonstrated through the implementation of the dynamic econometric APGAR model of prematurity risk. Smoking and alcohol consumption, facilitated by lack of education, have a significant impact on the health of newborns. Addictive behaviors increase the risk to fetal health and negatively affect the APGAR score, contributing to the poor condition of the newborn immediately after birth. Hypertension and diabetes have different effects on fetal health. While diabetes does not significantly influence the APGAR score in the short term, hypertension acts as an immediate risk factor. However, in some cases, medical interventions have mitigated the negative impact of hypertension on the health of newborns. The research emphasizes the need for specific interventions in health education and access to prenatal care for adolescent pregnant women, especially in disadvantaged environments, to reduce the risks associated with preterm births and fetal health.

The strengths of this study lie in its comprehensive approach to examining preterm births among adolescent pregnant women, a demographic often underrepresented in scientific literature. By focusing on individuals under the age of 18, the research provides valuable insights into a vulnerable population that faces heightened risks due to physiological immaturity and socioeconomic challenges. This focus allows for a nuanced understanding of the interplay between age-specific vulnerabilities and adverse neonatal outcomes. The study is further distinguished by its holistic consideration of exogenous and endogenous factors. By integrating variables such as educational attainment, lifestyle behaviors (smoking and alcohol consumption), and environmental conditions with medical comorbidities like diabetes and hypertension, the research captures the multifaceted nature of risk factors influencing preterm birth. This comprehensive analysis offers a more complete picture of the determinants of neonatal health, paving the way for targeted interventions. A significant strength of the study lies in its post-pandemic context. By utilizing data from 2022 to 2023, the research explores how the lingering effects of the COVID-19 pandemic have influenced maternal and neonatal health. This temporal specificity adds a layer of relevance to the findings, particularly in light of the pandemic’s disproportionate impact on vulnerable populations. Methodologically, the study employs robust statistical analyses, including multiple linear regression models, which enhance the reliability and interpretability of the results. The innovative application of an inverted APGAR scoring scale underscores the study’s commitment to advancing the assessment of neonatal outcomes, providing a unique and practical tool for clinical and public health use. Ethical considerations were rigorously upheld, with anonymized data collection and stringent inclusion and exclusion criteria ensuring the validity and reproducibility of the findings. These measures not only safeguard the integrity of the research but also reinforce its credibility in addressing sensitive health topics.

The study offers actionable insights into public health policy and practice. By emphasizing the critical role of education, access to healthcare, and socioeconomic support for adolescent mothers, the findings provide a foundation for developing targeted interventions aimed at mitigating preterm birth risks and improving neonatal health outcomes in disadvantaged communities. This alignment with real-world applications underscores the practical significance of the study’s contributions.

## Figures and Tables

**Table 1:** Descriptive statistics for the clinical samples regarding post-pandemic preterm births in the South-East region of Romania (observational group P and target group E).

Group		Structural Observations			Studies	Exogenous Factors			Endogenous Factors		Neonatal Health Outcomes		
		Age	EnvUR	TypeB		Adherence to			Incidence		APGAR5	APGAR10	APGAR20
						Smoking	Alcohol	Vices	DM	HBP			
Maximum Number of Options ^√^		3	2	2	5	2	2	2	2	2	10	10	10
P (231 preterm births)	Median option, the mean, and the significance errors of mean ^√√^	19–39 years 2.06 ***	Urban 1.46 **	Cesarean 1.52 **	9–12 cls 2 **	19% 1.19 **	20% 1.2 **	17% 1.17 **	16% 1.16 **	21% 1.21 **	7.85 3.15 **	7.95 3.05 **	7.99 3.01 **
E (54 preterm births of mothers under 18 years old)		12–18 years 1.00 ***	Rural 1.54 **	Cesarean 1.61 **	1–8 cls 3.39 **	57% 1.57 **	31% 1.31 **	20% 1.2 **	19% 1.19 **	44% 1.44 **	7.76 3.24	7.72 3.28	7.7 3.3
Total (3639 births in the Braila maternity hospital)		19–39 years 1.94 ***	Urban 1.47 ***	Cesarean 1.65 ***	9–12 cls 2.26 ***	27% 1.27 ***	27% 1.27 ***	24% 1.24 ***	9% 1.09 ***	11% 1.11 ***	7.83 3.17 **	7.91 3.09 **	7.93 3.07 **

Age—age (in years); EnvUR—living environment; TypeB—type of childbirth; Studies—education level; Smoking—tobacco smoker; Alcohol—alcohol consumer; Vices—vices (smoking + alcohol); DM—diabetes mellitus; HBP—high blood pressure; APGAR5—APGAR score (5 min); APGAR10—APGAR score (10 min); APGAR20—APGAR score (20 min). ^√^ Maximum number of options for data classification ^√√^ Significance errors of mean (ME): ***—ME < 1%; **—ME < 5%.

**Table 2:** The model equations.

Group P Equations		Group E Equations	
$\begin{array}{cc} \mathrm{APGAR}5.\;\mathrm{Group} & P\\ & =-5.435\;*\;\mathrm{Smoking}+4.021\\ & *\;\mathrm{Alcohol}+0.081\;*\;\mathrm{Vices}-0.381\\ & *\;\mathrm{DM}+0.158\;*\;\mathrm{HBP}+4.954 \end{array}$	(1)	$\begin{array}{cc} \mathrm{APGAR}5.\;\mathrm{Group} & E\\ & =-0.500\;*\;\mathrm{Smoking}+2.187\\ & *\;\mathrm{Alcohol}-2.254\;*\;\mathrm{Vices}-2.839\\ & *\;\mathrm{DM}+2.980\;*\;\mathrm{HBP}+2.927 \end{array}$	(2)
$\begin{array}{cc} \mathrm{APGAR}10.\;\mathrm{Group} & P\\ & =-5.512\;*\;\mathrm{Smoking}\;+4.050\\ & *\;\mathrm{Alcohol}+0.175\;*\;\mathrm{Vices}-0.512\\ & *\;\mathrm{DM}+0.330\;*\;\mathrm{HBP}+4.746 \end{array}$	(3)	$\begin{array}{cc} \mathrm{APGAR}10.\;\mathrm{Group} & E\\ & =-0.500\;*\;\mathrm{Smoking}+4.105\\ & *\;\mathrm{Alcohol}-4.237\;*\;\mathrm{Vices}-2.316\\ & *\;\mathrm{DM}+2.395\;*\;\mathrm{HBP}+3.053 \end{array}$	(4)
$\begin{array}{cc} \mathrm{APGAR}20.\;\mathrm{Group} & P\\ & =-5.535\;*\;\mathrm{Smoking}+4.008\\ & *\;\mathrm{Alcohol}+0.271\;*\;\mathrm{Vices}-0.402\\ & *\;\mathrm{DM}+0.307\;*\;\mathrm{HBP}+4.573 \end{array}$	(5)	$\begin{array}{cc} \mathrm{APGAR}20.\;\mathrm{Group} & E\\ & =-0.500\;*\;\mathrm{Smoking}+4.272\\ & *\;\mathrm{Alcohol}-4.404\;*\;\mathrm{Vices}-2.316\\ & *\;\mathrm{DM}+2.395\;*\;\mathrm{HBP}+3.053 \end{array}$	(6)

APGAR5. Group E, APGAR5. Group P, APGAR10. Group E, APGAR10. Group P, APGAR20. Group E, and APGAR20. Group P are the dependent variables of the models and represent the APGAR score at 5 min, 10 min, or 20 min after birth for observational group P and target group E. Smoking, Alcohol, Vices, DM, and HBP are the independent variables of the models in terms of each analyzed group.

**Table 3:** Model summary (representativity and significance tests).

Model ^a,b^	Pearson Coefficient (R)	Coefficient of Determination R^2^	Std. Error of the Estimate	Change Statistics				
				Change in Predictive Power	F Change	Degree of Freedom		Sig. F Change ^c^
						1	2	
APGAR5.LotE	0.697	0.486	1.884	0.486	9.093	5	48	***
APGAR5.LotP	0.528	0.278	1.098	0.278	17.363	5	225	***
APGAR10.LotE	0.783	0.614	1.704	0.614	15.250	5	48	***
APGAR10.LotP	0.561	0.314	0.996	0.314	20.620	5	225	***
APGAR20.LotE	0.782	0.612	1.751	0.612	15.130	5	48	***
APGAR20.LotP	0.491	0.241	1.171	0.241	14.321	5	225	***

^a^ Independent variables: (Constant), HBP (High blood pressure), DM (Diabetes mellitus), Vices (adherence to vices), Smoking (Tobacco smokers), Alcohol (Alcohol consumers). ^b^ Dependent variables APGAR score at 5 min, 10 min, or 20 min after birth for observational group P or target group E (for each model, statistics are based only on cases for which Group = E or Group = P). ^c^ *** High statistical significance (Sig. F < 0.005).

**Table 4:** Analysis of variance statistical test (ANOVA).

Model ^a,b^	Sum of Squares			df			Mean Square		F	Sig. F ^d^
	Rg ^c^	Rs ^c^	T ^c^	Rg ^c^	Rs ^c^	T ^c^	Rg	Rs		
APGAR5. Group E	161.438	170.433	331.870	5	48	53	32.288	3.551	9.093	***
APGAR10. Group E	221.439	139.395	360.833	5	48	53	44.288	2.904	15.250	***
APGAR20. Group E	232.031	147.228	379.259	5	48	53	46.406	3.067	15.130	***
APGAR5. Group P	104.602	271.095	375.697	5	225	230	20.920	1.205	17.363	***
APGAR10. Group P	102.246	223.131	325.377	5	225	230	20.449	0.992	20.620	***
APGAR20. Group P	98.248	308.713	406.961	5	225	230	19.650	1.372	14.321	***

^a^ Independent variables: (Constant), HBP (High blood pressure), DM (Diabetes mellitus), Vices (adherence to vices), Smoking (Tobacco smokers), Alcohol (Alcohol consumers). ^b^ Dependent variables APGAR Score at 5 min, 10 min, or 20 min after birth for observational group P or target group E (for each model, statistics are based only on cases for which Group = E or Group = P). ^c^ Rg = regression results, Rs = residual results, T = total results. ^d^ *** High statistical significance (Sig. F < 0.005).

**Table 5:** Pearson correlation coefficients.

Indicator ^a^	Group	APGAR5	APGAR10	APGAR20	EnvUR	Studies	TypeB	Smoking	Alcohol	Vices	DM	HBP	PREMATURE
APGAR5	Group E												
	Group P												
APGAR10	Group E	0.958 ***											
	Group P	0.962 ***											
APGAR20	Group E	0.947 ***	0.996 ***										
	Group P	0.909 ***	0.939 ***										
EnvUR	Group E	−0.209 *	−0.202 *	−0.19 *									
	Group P	0.367 ***	0.311 ***	0.325 ***									
Studies	Group E	−0.241 **	−0.18 *	−0.168	0.234 **								
	Group P	−0.328 ***	−0.304 ***	−0.311 ***	−0.529 ***								
TypeB	Group E	0.399 ***	0.394 ***	0.39 ***	−0.741 ***	−0.182 *							
	Group P	−0.417 ***	−0.367 ***	−0.389 ***	−0.896 ***	0.516 ***							
Smoking	Group E	0.114	0.151	0.153	−0.124	0.377 ***	0.158						
	Group P	−0.421 ***	−0.421 ***	−0.37 ***	−0.447 ***	0.583 ***	0.396 ***						
Alcohol	Group E	0.401 ***	0.436 ***	0.436 ***	−0.65 ***	0.026	0.541 ***	0.584 ***					
	Group P	−0.34 ***	−0.332 ***	−0.291 ***	−0.416 ***	0.574 ***	0.367 ***	0.973 ***					
Vices	Group E	0.025	−0.054	−0.057	−0.545 ***	0.134	0.403 ***	0.436 ***	0.746 ***				
	Group P	−0.298 ***	−0.292 ***	−0.248 ***	−0.369 ***	0.566 ***	0.314 ***	0.87 ***	0.904 ***				
DM	Group E	−0.181 *	−0.199 *	−0.197 *	−0.513 ***	0.17	0.38 ***	0.025	0.293 **	0.469 ***			
	Group P	−0.173 ***	−0.21 ***	−0.157 **	0.131 **	0.047	−0.116 **	0.156 **	0.144 **	0.189 ***			
HBP	Group E	0.439 ***	0.437 ***	0.434 ***	−0.814 ***	−0.084	0.714 ***	0.243 **	0.758 ***	0.565 ***	0.533 ***		
	Group P	−0.186 ***	−0.148 **	−0.133 **	−0.457 ***	0.252 ***	0.431 ***	0.476 ***	0.457 ***	0.332 ***	0.215 ***		
PREMATURE	Group E	0.953 ***	0.878 ***	0.871 ***	−0.285 **	−0.313 **	0.429 ***	0.068	0.372 ***	0.031	−0.068	0.502 ***	
	Group P	0.857 ***	0.829 ***	0.81 ***	0.319 ***	−0.283 ***	−0.379 ***	−0.188 ***	−0.116 **	−0.089 *	−0.164 **	−0.065	

^a^ APGAR5—APGAR score (5 min); APGAR10—APGAR score (10 min); APGAR20—APGAR score (20 min); EnvUR—living environment; Studies—education level; TypeB—type of childbirth; Smoking—tobacco smoker; Alcohol—alcohol consumer; Vices—vices (smoking + alcohol); DM—diabetes mellitus; HBP—high blood pressure; PREMATURE—birthweight (see also Appendix A). Significance levels: *: *p* < 0.05; **: *p* < 0.01; ***: *p* < 0.001.

## Data Availability

The data that support the findings of this study are available from the corresponding author upon request.

## Appendix A. Indicators

**Indicator Symbol**	**Indicator Description**	**Low Risk High Risk** **Option (*)**														
Year	Year	2022 (1)									2023 (1)					
Age	Age (in years)	12–18 (1)				19–39 (2)								>40 (3)		
EnvUR	Living environment	Urban (1)								Rural (2)						
Studies	Education level	Higher education (1)			Highschool (2)			Middle school (3)					No studies (4)		Not mentioned (5)	
TypeB	Type of childbirth	Vaginal birth (1)							Cesarean section (2)							
BPREM	Preterm labor/delivery/birth	No (1)							Yes (2)							
Smoking	Tobacco smoker	No (1)							Yes (2)							
Alcohol	Alcohol consumer	No (1)							Yes (2)							
Vices	Vices (Smoking + alcohol)	No (1)							Yes (2)							
DM	Diabetes mellitus	No (1)							Yes (2)							
HBP	High blood pressure	No (1)							Yes (2)							
PREMATURE	Birthweight	GR I 2000–2500 g (1)			GR II 1500–2000 g (2)				GR III 1000–1500 g (3)						GR IV < 1000 g (4)	
APGAR5	APGAR score (5 min)	10 (1)		9 (2)	8 (3)	7 (4)	6 (5)		5 (6)			4 (7)		3 (8)	2 (9)	1 (10)
APGAR10	APGAR score (10 min)	10 (1)		9 (2)	8 (3)	7 (4)	6 (5)		5 (6)			4 (7)		3 (8)	2 (9)	1 (10)
APGAR20	APGAR score (20 min)	10 (1)		9 (2)	8 (3)	7 (4)	6 (5)		5 (6)			4 (7)		3 (8)	2 (9)	1 (10)
(*)—the options number: 1 up to 10.																
